# A Scoping Review of Non-Structural Airway Disease as a Cause of Poor Performance in Racehorses

**DOI:** 10.3390/ani13030429

**Published:** 2023-01-27

**Authors:** Ann Cullinane, Marie Garvey, Cathal Walsh, James Gibbons, Alan Creighton

**Affiliations:** 1 The Irish Equine Centre, Johnstown, Naas, Co., W91 RH93 Kildare, Ireland; 2 Department of Mathematics and Statistics, University of Limerick, V94 T9PX Limerick, Ireland

**Keywords:** racehorse, Thoroughbred, poor performance, respiratory disease, review, equine asthma

## Abstract

**Simple Summary:**

The association between poor performance and respiratory disease in Thoroughbred racehorses that do not have a structural abnormality of the respiratory tract is often based on anecdotal evidence. We examined peer reviewed publications to determine if there was scientific evidence to link conditions such as inflammation of the airways, asthma, tracheal mucous, and exercise-induced pulmonary haemorrhage (EIPH) with decreased athletic performance. This is a complex field and studies have yielded conflicting results as to the impact of such conditions on performance. For example, some investigators found a significant association between poor racing performance and low-grade EIPH and mild to moderate asthma. In contrast, others have suggested that they may represent a normal response to training and the stabling environment. The key outcome of the review was that there is a dearth of studies unequivocally linking non-structural airway disease with poor performance, and there is a lack of an internationally harmonised approach to the assessment of racehorse performance. Improved investigations providing better quality evidence would facilitate comparison across studies, increase our understanding of the conditions associated with poor performance, safeguard horse welfare, and assist trainers to achieve their goals.

**Abstract:**

The association between poor performance and respiratory disease in Thoroughbred racehorses that do not have a structural abnormality of the respiratory tract, is often based on anecdotal evidence. The objective of this scoping review was to examine the scientific evidence for such associations. Publications were selected based on a search of three databases (PubMed, Scopus, and CAB Direct), in English and without date restriction, followed by a screening process to exclude non-relevant papers, duplicates, and reviews. This process identified 996 publications of which 20 were analysed using the Quality in Prognosis Studies (QUIPS) tool. The results indicated that the evidence supporting the relationship between proposed diagnostic indicators and poor performance is variable. There is a need for better quality evidence. In particular, there are conflicting reports relating to the impact of equine asthma and EIPH on athletic performance. Furthermore, a lack of standardisation in the measurement of racehorse performance makes it difficult to compare findings from different studies. The industry would benefit from high-level guidance concerning the design of controlled performance studies in Thoroughbred racehorses to collect comprehensive data and facilitate targeted interventions.

## 1. Introduction

Respiratory disease has been cited as a very common cause of a loss of training days in Thoroughbred racing yards [1,2]. Although it is reasonable to presume that optimal airway function is necessary for horses to perform at their best on the racetrack, there appears to be a dearth of studies investigating the impact of respiratory disease, which does not necessitate surgical intervention, on exercise intolerance and poor performance. There is no standardised method of assessing poor performance, and the use of a wide variety of different performance measures has made it very difficult to compare the data generated by different studies. In contrast, there is a large body of scientific work devoted to the detection, epidemiology, and aetiopathogenesis of both subclinical and clinical airway infections [3,4]. Inflammatory airway disease (IAD) is common in racehorses and significant efforts have been made to define this disease [5], not always without controversy [6,7]. Recurrent air-way obstruction (RAO), a condition primarily observed in older horses, and IAD represent a spectrum of chronic inflammatory disease of the airways in horses that resembles human asthma. The most recent consensus statement for IAD defines the condition as mild to moderate equine asthma [5]. Thus, the term IAD has been generally replaced with asthma, as it is easily understood by horse owners and facilitates international dialogue on the disease. Both terms are used in this review as the majority of the publications identified and discussed here predate the new classification. The consensus statement describes the diagnosis of IAD or mild to moderate asthma, as based on factors such as poor performance, cough, mucous detection on endoscopy, bronchoalveolar lavage (BAL) cytology, and abnormal lung function. However, this definition has not met with universal support and there are conflicting reports regarding the impact of asthma and other respiratory disorders on athletic performance [6,7,8,9,10]. Similarly, although there is a consensus statement for exercise-induced pulmonary haemorrhage (EIPH), there is debate as to whether it impairs racing performance in Thoroughbred racehorses or if it is simply reflective of strenuous athletic effort [11]. In contrast, there are also concerns about the impact of EIPH on the well-being of racehorses and its role in sudden deaths [12].

It appears that the association of poor performance with respiratory disease in Thoroughbred racehorses that do not have a structural abnormality of the respiratory tract, often relies heavily on veterinary clinician/trainer experience and anecdotal evidence. This is not to suggest an absence of an association between airway issues and performance, rather than to safeguard horse welfare, there is a need for evidence-based practice. Athletes need to maintain healthy airways for optimal performance and targeted interventions to minimise the potential for mild conditions progressing to clinical problems. The objective of this review was to examine the evidence of association between poor performance and non-structural respiratory disease in Thoroughbred racehorses in the peer reviewed literature.

## 2. Materials and Methods

A scoping review was carried out based on the Preferred Reporting Items for Systematic reviews and Meta-Analyses extension for Scoping Reviews (PRISMA-ScR) [13]. A study protocol was not filed. All peer-reviewed studies of both flat and jump Thoroughbred racehorses published in English were eligible. Publications relating to Standardbreds, sport horses, and other equine breeds were excluded. The search did not include any limits with respect to publication date.

The NIH Pubmed, SCOPUS, and CAB direct databases were searched for studies between September and November 2022. The search of NIH Pubmed and SCOPUS databases was carried out on the 30th September 2022 using the following terms: ((thoroughbred [Title/Abstract]) AND (racehorses [Title/Abstract])) AND performance [Ti-tle/Abstract]) and TITLE-ABS-KEY (thoroughbred AND racehorses AND performance), respectively. A search of the CAB direct database was carried out on 28th November 2022 using the search string: (ab:(thoroughbred) OR title:(thoroughbred)) AND (ab:(racehorses) OR title:(racehorses)) AND (ab:(performance) OR title:(performance)).

The results obtained from each database were exported to Endnote X7 and subsequently imported to Rayyan QCRI systematic review web application [14], where replicates were excluded. Two reviewers (AC* and MG) screened the remaining publications together by title and abstract using the following exclusion criteria: not referring to respiratory disease; not referring to poor performance; referring to respiratory disease due to structural abnormalities; referring to medication or vaccination; diagnosis; review article; not in the English language; and conference proceedings. Reference lists of previous reviews were hand searched for additional full texts not identified by the searches. After this selection, a full text review was conducted by one of the reviewers (A.C.*) to select the publications for inclusion. Three reviewers (A.C.*, M.G., and C.W.) evaluated the quality of the included studies using the Quality in Prognosis Studies (QUIPS) modified tool [15]. The QUIPS tool assesses study quality under six domains: study participation; study attrition; prognostic factor measurement; outcome measurement; study confounding; statistical analysis and reporting. Each domain was rated as having a low, moderate, or high risk of bias using a standardised form provided on the Cochrane website [16]. Results were summarised in a stacked bar chart for each domain.

Data charting was performed in Excel by one reviewer (A.C.*) and checked independently by a second reviewer (M.G.). The following data were extracted from each review: author and publication date, primary focus in relation to performance, centre, study design, number of Thoroughbred horses, racing event (flat or jump), status, performance metrics, endoscopy, tracheal wash (T.W.), BAL, bacterial culture, environment, blood gas analysis, miscellaneous, and statistics. Performance metrics were categorised by one reviewer (J.G.) into the following eight categories and data extraction was reviewed by M.G.:

- Horse or race speed/time-based measures.
- Measures based on race earnings.
- Measures based on finishing position within race.
- Categorical (yes/no) measures of starting, winning and/or placing in races.
- Count measures of number of starts, wins and/or places.
- Measures of career duration (in days).
- Time to (return to) racing (in days).
- No objective measure of performance.

## 3. Results

### 3.1. Search Results

**Figure 1:**
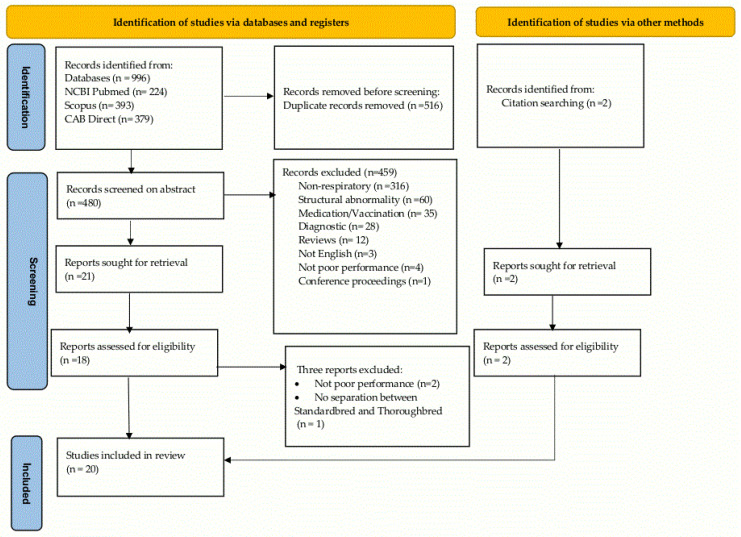
Search and selection flowchart for scoping review of non-structural airway disease as a cause of poor performance in racehorses [17].

Based on the search of the three databases, 996 publications were identified (see Figure 1 [17]). Of these, 516 replicates were removed. The titles and abstracts of 480 were read to assess their suitability for inclusion based on the exclusion criteria, and 459 were excluded. The remaining twenty-one articles were read in their entirety to ensure that they met the inclusion criteria, and a further three were removed as two did not include poor performance and one did not separate their findings relating to Thoroughbred racehorses from Standardbred horses. The characteristics of the twenty studies retained, which includes two additional publications identified from the reference list of the reviewed papers, are summarised in Table 1, Table 2 and Table 3 below.

The majority of the studies identified (n = 9) concerned investigations relating to airway inflammation, i.e., equine asthma formerly known as IAD [10,18,19,20,21,22,23,24,25]. Results described included BAL [18,20,21,22,24] and TW findings [19,20,23,25], tracheal mucus [10,20,23,24,25], and pharyngeal lymphoid hyperplasia (PLH) [10,19,23,24] observed by endoscopy. EIPH was the primary focus of five studies [26,27,28,29,30]. Three studies were focussed on respiratory agents, i.e., viruses (n= 2) [31,32] and pollutants (n = 1) [33] as potential contributing factors to poor performance. Two studies related to the return to racing after recovery from pleuropneumonia and pulmonary abscesses [34,35]. One study described the endoscopic findings, the environment, and the racing career of horses presenting coughs or poor performance [36].

Four studies did not use any objective measure of performance; the horses selected for inclusion in each had a history of poor performance [18,19,20,21]. Measures based on categorical measures of starts/wins/places were most frequently used (n = 8) [22,23,24,26,30,32,35,36], while measures based on earnings [10,26,28,29,30,34] and on finishing position [23,24,25,26,29,30] were used in six studies. In contrast, time to return to racing was used in only one study [36]. A number of studies utilised several individual measures from within the same category (e.g., finishing position in race and earnings), while ten studies used measures from at least two categories [10,23,24,26,27,28,29,30,34,36]. One study used measures from three separate categories and assessed these over two distinct time periods: lifetime racing career and career after time of examination [28].

### 3.2. Quality Appraisal

**Figure 2:**
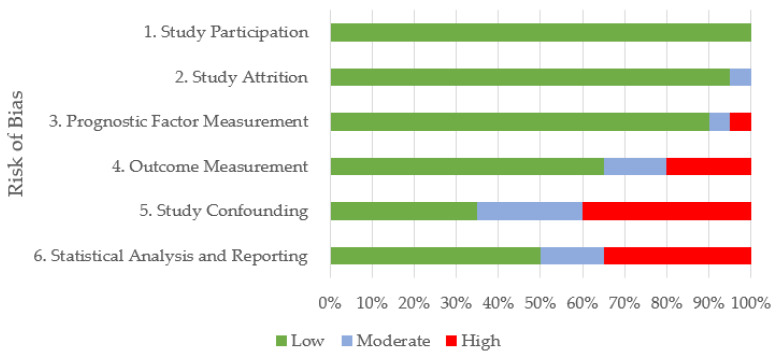
Summary of quality appraisal for the 20 reviewed publications using the Quality in Prognosis Studies (QUIPS) tool. Quality was assessed as having low, moderate, or high risk of bias across six domains.

The proportions of studies with a low, moderate, or high risk of bias under the six domains across all 20 publications are summarised in Figure 2. A high risk of bias related predominantly to study confounding (40%) and statistical analysis and reporting (35%). In 13 articles, important potential confounders were not appropriately accounted or adjusted for, resulting in moderate to high potential bias with respect to the relationship between prognostic factor and performance outcome. In 50% of the studies assessed, the risk of bias in relation to statistical analysis and reporting was moderate or high. Factors attributing to this included the rationale for the statistical tests carried out being unclear or inappropriate to answer the key questions of interest, or only simple descriptive statistics being used. Conversely, the risk of bias in terms of study participation was low across all 20 studies.

The risk of bias in individual studies is reported in Table 1. Fifty six percent of the studies focused on the investigation of airway inflammation had high bias in relation to both study confounding and statistical analysis and reporting. They included investigations of horses with poor performance without comparison to horses performing satisfactorily. Only one study that focused on EIPH had high bias [27]. This was a large retrospective cross-sectional study of racehorses where the confounding between the EIPH status and the outcome measures was not adjusted for in the analysis. Similarly, the potential bias due to unmeasured confounders in one longitudinal cohort study investigating respiratory viruses was high [32]. The two studies related to prognosis after recovery from pleuropneumonia and pulmonary abscesses had moderate bias in relation to attrition (n = 1) and study confounding and statistical analysis (n = 1) [34,35]. An investigation of coughing horses had high bias in relation to the outcome measurement, confounding, and analysis [36].

**Table 1:** Quality appraisal for the 20 reviewed publications using the Quality in Prognosis Studies (QUIPS) tool. Quality was assessed as having low, moderate, or high risk of bias across six domains.

		Risk of Bias					
Focus	Study	1. Study Participation	2. Study Attrition	3. Prognostic Factor Measurement	4. Outcome Measurement	5. Study Confounding	6. Statistical Analysis and Reporting
EA	Fogarty and Buckley 1991 [22]	Low	Low	Low	Low	Moderate	Moderate
EA	Holcombe et al. 2006 [23]	Low	Low	Moderate	Low	Low	Low
EA	Ivester et al. 2018 [24]	Low	Low	Low	Low	Low	Low
EA	Salz et al. 2016 [25]	Low	Low	Low	Low	Moderate	Low
EA	McKane et al. 1995 [21]	Low	Low	Low	Moderate	High	High
EA	Nolen- Walston et al. 2013 [18]	Low	Low	Low	Moderate	High	High
EA	Saulez and Gummow 2009 [10]	Low	Low	Low	Low	High	High
EA	Allen et al. 2006 [20]	Low	Low	Low	High	High	High
EA	Kusano et al. 2008a [19]	Low	Low	Low	High	High	High
P	Ainsworth et al. 2000 [34]	Low	Moderate	Low	Low	Low	Low
p	Seltzer et al. 1996 [35]	Low	Low	Low	Low	Moderate	Moderate
EIPH	Crispe et al. 2017 [26]	Low	Low	Low	Low	Low	Low
EIPH	Hinchcliff et al. 2005 [29]	Low	Low	Low	Low	Low	Low
EIPH	Morley et al. 2015 [30]	Low	Low	Low	Low	Low	Low
EIPH	Sullivan et al. 2015 [28]	Low	Low	Low	Moderate	Moderate	Low
EIPH	Preston et al. 2015 [27]	Low	Low	High	High	High	High
RA	Couetil et al. 2021 [31]	Low	Low	Low	Low	Low	Low
RA	Araneda and Cavada 2022 [33]	Low	Low	Low	Low	Moderate	Moderate
RA	Back et al. 2019 [32]	Low	Low	Low	Low	High	Low
C	Kusano et al. 2008b [36]	Low	Low	Low	High	High	High

Abbreviations: C = coughing, EA = Equine asthma, EIPH = exercise-induced pulmonary haemorrhage, P = pleuropneumonia and pulmonary abscesses, and RA = respiratory agents.

**Table 2:** Study Characteristics.

Authors	Country	Primary Focus in Relation to Performance	Centre	Study Design	No. of TB Horses	Flat	Jump	Status	Performance
Ainsworth et al. 2000 [34]	USA/ Canada	Pulmonary abscesses: Diagnostic radiographs and ultrasonographic images described and affected regions identified.	Four hospitals.	Retrospective cohort.	20	U	U	Medical records of a primary lung abscess.	Y- ii and v (no. of starts and earnings per start preceding and following the horses illness)
Allen et al. 2006 [20]	UK	IAD: Three different case definitions of disease are used. Tracheal mucus scored from 0–3. >20% neutrophils in TW. >5% neutrophils in BAL.	One referral clinic.	Retrospective case series.	91		Y	Referred for investigation of poor athletic performance.	viii
Araneda and Cavada 2022 [33]	Chile	Atmospheric pollutants (PM10, PM2.5, O3, NO2, NO, SO2, and CO), humidity, and temperature.	One racetrack.	Correlational observational.	162	Y		Race winners.	Y- i
Back et al. 2019 [32]	Ireland	Equine rhinitis virus seroconversion detected by complement fixation test.	One yard.	Longitudinal cohort.	30	Y		In training.	Y- iv
Couetil et al. 2021 [31]	USA	Equine herpesviruses and equine rhinitis viruses. Virus-specific qPCR.	Five barns.	Prospective observational.	31	Y		Racing.	Y- i
Crispe et al. 2017 [26]	Australia	EIPH: tracheobronchoscopic examinations graded 0-4 using a referenced scoring system.	Three racetracks.	Observational cross- sectional.	1567	Y		Racing.	Y- i, ii, iii, and iv
Fogarty and Buckley 1991 [22]	Ireland	BAL: neutrophil %, haemosiderophage %, TCFU. Lower airway infection was characterised by a TCFU >40, neutrophil % > 10%, in association with toxic cellular changes, cellular clumping, and increased mucus production. EIPH detection was based on the presence of haemosiderophages with or without intra- or extracellular red blood cells.	One laboratory.	Case control.	65 + 11 controls	U	U	Total of 65 with severe exercise intolerance during strenuous exercise and 11 having competed successfully.	Y- iv
Hinchcliff et al. 2005 [29]	Australia	EIPH: tracheobronchoscopic examinations graded 0–4 using a referenced scoring system.	Four racetracks.	Cross- sectional.	744	Y		Racing.	Y- ii and iii
Holcombe et al. 2006 [23]	USA	Tracheal mucus grade (0–4) as described by Gerber et al. 2004 [37], PLH grade (0–4) as described by Raker and Boles 1978 [38] and TW turbidity and differential cytology counts.	One racetrack.	Longitudinal cohort.	327	Y		Racing.	Y- iii and iv
Ivester et al. 2018 [24]	USA	BAL differential cytology counts. Mild asthmatics were defined as horses with >5% neutrophils, >2% mast cells, or >1% eosinophils, or any combination thereof. Respirable and inhalable dust, respirable endotoxin, and respirable β-glucan exposure measurements were obtained within one week after racing.	Three race meets.	Prospective cohort.	64	Y		Racing.	Y- i, iii, and iv
Kusano et al. 2008a [19]	Japan	Assessment of horses IAD vs. non-IAD. Tracheal aspirates containing >20% neutrophils were considered indicative of IAD.	One training facility.	Cross- sectional.	76	Y		Presenting with cough or poor performance.	viii
Kusano et al. 2008b [36]	Japan	Tracheal mucus grades (0–3), tracheal aspirate cytology (> 20% neutrophils diagnostic for IAD) SAA, Fbg and SP-D measurements from coughing vs. non-coughing horses.	One Training facility.	Cross- sectional.	95 (86 + 9 controls)	Y		Total of 86 presenting with cough or poor performance and 9 controls without respiratory abnormality.	Y- iv, v, and vii
McKane et al. 1995 [21]	Australia	BAL cytology (differential cell counts including neutrophil, erythrocyte, and haemosiderophage percentage and the total nucleated cell concentration) and oxygen saturation of haemoglobin in arterial blood.	One referral clinic.	Case series.	24	U	U	Reported poor racing performance.	viii
Morley et al. 2015 [30]	South Africa	EIPH: tracheobronchoscopic examinations graded 0-4 using a referenced scoring system.	Five racetracks.	Prospective cross- sectional.	886	Y		Racing.	Y- ii, iii, and iv
Nolen- Walston et al 2013 [18]	USA	IAD (now referred to as asthma) subtypes: eosinophilic-mastocytic: ≥0.5% eosinophils, ≥ 2% mast cells or both. Neutrophilic, ≥ 5% neutrophils, and Mixed ≥ 5%.neutrophils as well as ≥ 0.5% eosinophils, ≥ 2% mast cells, or all three.	One referral clinic.	Retrospective case series.	45	Y (42)	Y (3)	Examined because of poor performance.	viii
Preston et al. 2015 [27]	Hong Kong	EIPH: tracheobronchoscopic examinations. Graded 0–4 using a referenced scoring system.	Three racetracks.	Retrospective cross sectional.	822	Y		In training.	Y- v and vi
Salz et al. 2016 [25]	Australia	Tracheal mucus grade (0–4), tracheal blood grade (0–4), and TW differential cytology counts >20% neutrophils was considered to be significant.	One yard.	Cross- sectional.	155	Y		In training.	Y- iii
Saulez and Gummow (2009) [10]	South Africa	Laryngeal function grade (1–4), PLH grade (1–4), and tracheal mucus grade (0–5), on the basis of referenced grading systems.	Five racetracks.	Prospective cross- sectional.	1005	Y		Racing.	Y- ii and v
Seltzer et al. 1996 [35]	USA	Pleuropneumonia-clinical signs, ultrasonography, and bacteriology.	One hospital.	Retrospective case series.	70	U	U	Medical records of pneumonia and pleural effusion	Y- iv Post treatment won≥ 1 race, had earnings, raced without earnings or did not race.
Sullivan et al. 2015 [28]	Australia	EIPH: tracheobronchoscopic examinations. Graded 0–4 using a referenced scoring system	Four racetracks.	Prospective longitudinal.	744	Y		Racing.	Y- ii, v, and vi

Abbreviations: BAL = bronchoalveolar lavage, EIPH = exercise-induced pulmonary haemorrhage, Fbg = fibrinogen, IAD = inflammatory airway disease, PLH= pharyngeal lymphoid hyperplasia, qPCR = quantitative polymerase chain reaction, SAA = serum amyloid A, SP-D = pulmonary surfactant protein D, TB = Thoroughbred, TCFU = total bacterial colon-forming units, TW = tracheal wash, U = unknown, and Y = Yes. (i) Horse or race speed/time-based measures. (ii) Measures based on race earnings. (iii) Measures based on finishing position within race. (iv) Categorical (yes/no) measures of starting, winning, and/or placing in races. (v) Count measures of number of starts, wins, and/or places. (vi) Measures of career duration (in days). (vii) Time to (return to) racing (in days). (viii) No objective measure of performance.

**Table 3:** Methodology.

Authors	Primary Focus in Relation to Performance	Endoscopy	TW	BAL	Bacterial Culture	Envir.	Blood Gas	Misc.	Statistics
Ainsworth et al. 2000 [34]	Pulmonary abscesses: Diagnostic radiographs and ultrasonographic images described and affected regions identified.				Y			Radiography	Wilcoxon rank sum, Wilcoxon signed ranks, and Fischer exact tests.
Allen et al. 2006 [20]	IAD: Three different case definitions of disease are used. Tracheal mucus scored from 0–3. >20% neutrophils in TW. >5% neutrophils in BAL.	Y	Y	Y	Y				Kappa statistics, Spearman rank correlations, and binomial logistic regression analysis.
Araneda and Cavada 2022 [33]	Atmospheric pollutants (PM10, PM2.5, O3, NO2, NO, SO2, and CO), humidity, and temperature.					Y			Pearson’s correlation co-efficient.
Back et al. 2019 [32]	Equine rhinitis virus seroconversion detected by complement fixation test.							Serology	Chi-squared test.
Couetil et al. 2021 [31]	Equine herpesviruses and equine rhinitis viruses. Virus-specific qPCR.	Y		Y		Y		qPCR	Generalised linear mixed models.
Crispe et al. 2017 [26]	EIPH: tracheobronchoscopic examinations graded 0-4 using a referenced scoring system.	Y							Linear mixed effects and multiple logistic regression models. Generalised estimating equations were used for analysis of binary responses.
Fogarty and Buckley 1991 [22]	BAL: neutrophil %, haemosiderophage %, TCFU. Lower airway infection was characterised by a TCFU >40, neutrophil % > 10%, in association with toxic cellular changes, cellular clumping, and increased mucus production. EIPH detection was based on the presence of haemosiderophages with or without intra- or extracellular red blood cells.	Y		Y	Y	Y			Student’s *t* test.
Hinchcliff et al. 2005 [29]	EIPH: tracheobronchoscopic examinations graded 0–4 using a referenced scoring system.	Y							Multi-variate logistic regression.
Ivester et al. 2018 [24]	BAL differential cytology counts. Mild asthmatics were defined as horses with >5% neutrophils, >2% mast cells, or >1% eosinophils, or any combination thereof. Respirable and inhalable dust, respirable endotoxin, and respirable β-glucan exposure measurements were obtained within one week after racing.	Y		Y		Y			Mixed logistic regression models, Spearman rank correlations, and Tukey’s post hoc analysis.
Kusano et al. 2008a [19]	Assessment of horses IAD vs. non-IAD. Tracheal aspirates containing >20% neutrophils were considered indicative of IAD.	Y	Y			Y			Fisher’s exact test.
Kusano et al. 2008b [36]	Tracheal mucus grades (0–3), tracheal aspirate cytology (>20% neutrophils diagnostic for IAD) SAA, Fbg and SP-D measurements from coughing vs. non-coughing horses.		Y					Inflammatory markers.	Wilcoxon signed ranks, Kruskal–Wallis, and Scheffe’s F tests.
McKane et al. 1995 [21]	BAL cytology (differential cell counts including neutrophil, erythrocyte, and haemosiderophage percentage and the total nucleated cell concentration) and oxygen saturation of haemoglobin in arterial blood.			Y			Y		Mann–Whitney U test and Spearman rank correlations.
Morley et al. 2015 [30]	EIPH: tracheobronchoscopic examinations graded 0-4 using a referenced scoring system.	Y							Multivariable logistic and linear regression models.
Nolen- Walston et al 2013 [18]	IAD (now referred to as asthma) subtypes: eosinophilic-mastocytic: ≥0.5% eosinophils, ≥ 2% mast cells or both. Neutrophilic, ≥ 5% neutrophils, and Mixed ≥ 5%.neutrophils as well as ≥ 0.5% eosinophils, ≥ 2% mast cells, or all three.	Y		Y			Y		Kruskal–Wallis, Spearman rank correlations, Fischer exact tests, and simple logistic regression.
Preston et al. 2015 [27]	EIPH: tracheobronchoscopic examinations. Graded 0–4 using a referenced scoring system.	Y							Kruskal–Wallis and Cox regression analysis.
Salz et al. 2016 [25]	Tracheal mucus grade (0–4), tracheal blood grade (0–4), and TW differential cytology counts >20% neutrophils was considered to be significant.	Y	Y						Ordinal logistic regression and Somer’s D (non-parametric).
Saulez and Gummow (2009) [10]	Laryngeal function grade (1–4), PLH grade (1–4), and tracheal mucus grade (0–5), on the basis of referenced grading systems.	Y							Wilcoxon rank sum, Mann–Whitney U test, Kruskal–Wallis, Chi-squared test, and regression analysis.
Seltzer et al. 1996 [35]	Pleuropneumonia-clinical signs, ultrasonography, and bacteriology.				Y			Ultrasonography.	Two sample z test.
Sullivan et al. 2015 [28]	EIPH: tracheobronchoscopic examinations. Graded 0–4 using a referenced scoring system	Y							Linear and negative binomial regression.

Abbreviations: BAL = bronchoalveolar lavage, EIPH= exercise-induced pulmonary haemorrhage, Envir. = Environment, Fbg = fibrinogen, IAD = inflammatory airway disease or asthma, Misc. = miscellaneous, PLH = pharyngeal lymphoid hyperplasia, SAA = serum amyloid A, SP-D = pulmonary surfactant protein D, TCFU = total bacterial colon-forming units, TW = tracheal wash, U = unknown, and Y = Yes.

### 3.3. Equine Asthma Formerly Inflammatory Airway Disease (IAD)

Of the nine studies focused on equine asthma and inflammation of the airways, only one had a low risk of bias [24], three had a moderate risk of bias [22,23,25], and five had a high risk of bias [10,18,19,20,21].

The simplest approach taken to investigate the association between equine asthma and poor performance has been the examination of horses and samples collected from horses, which were referred to clinics, i.e., observational studies without controls. A study by Allen et al. [20] indicated that subclinical IAD was a common finding in National Hunt (NH) horses referred to a Sports Medicine Centre in the UK, with a history of poor athletic performance. In the cytological analysis of TW and BAL fluid they found that 70% of the horses had evidence of neutrophilic inflammation, and on endoscopy after exercise, 68% had some tracheal mucus. As no control horses were examined, the authors state it was not possible to draw conclusions regarding the effect of these findings on performance. Furthermore, 87% of the horses had some form of upper respiratory tract obstruction, which may have impaired their performance. Kusano et al. [19] performed a similar investigation without control horses, at a Japan Racing Association (JRA) training facility. Seventy-six Thoroughbred racehorses presenting coughs or poor performance were investigated by an endoscopic examination and TW at the JRA racehorse hospital. No information was provided in relation to when the examinations were performed relative to exercise. Seventy-three per cent had TWs in excess of 20% neutrophils on a differential cell count. These horses were classified as having IAD and the remaining 27% were classified as non-IAD. Upper airway abnormalities, such as laryngeal hemiplegia and dorsal displacement of the soft palate, were observed in these horses, but there was no significant difference in the incidence in the IAD and non-IAD groups [19].

Fogarty and Buckley [22] compared BAL findings in 11 control horses racing successfully and 65 horses that had presented respiratory disease and exercise intolerance. The latter had previously raced successfully, which limited the impact of the potential confounding variables. They presented a history of loss of momentum or stopping during strenuous exercise, respiratory distress post exercise, occasional coughing, and nasal discharge. Endoscopic examination of nine horses that raced successfully and 47 with exercise intolerance, found no mucopus in the former but moderate or copious amounts in 31% of the latter. On the examination of BAL fluids, the percentages of neutrophils, haemosiderophages, and bacterial colony-forming units were significantly higher in the poorly performing group. The authors conclude that these differences, combined with the subsequent performance of 36 of the 65 horses after following recommendations based on the BAL findings, suggest an association between the findings and poor performance. The recommendations included antibiotic treatment specific to the organisms isolated and environmental measures such as alteration in bedding, feeding practices, and ventilation.

McKane et al. [21] evaluated 24 horses performing below trainers’ expectations through clinical exercise testing on a high-speed treadmill and reported an inverse relationship between hypoxia and the total nucleated cell count in BAL fluid. Nolen-Watson et al. [18] retrospectively examined BAL cytology data including IAD subtypes (eosinophilic-mastocytic, neutrophilic, and mixed) from 98 horses (45 Thoroughbreds) referred for poor performance, of which 19 had non-inflammatory BAL fluid and were designated controls. All horses were evaluated by a high-speed treadmill test and in contrast to the McKane et al. study, no association between BAL cytology and pulmonary gas exchange was identified.

Other larger scale studies incorporated performance measurements at the racetrack and statistical methods to control for potential confounding variables. A longitudinal study by Holcombe et al. [23] of horses considered healthy enough to race, examined if tracheal mucus and large airway inflammation were associated with poor racing performance at a racetrack in the USA. Racing records within two weeks of endoscopic examination and the collection of a TW were examined. Horses were examined monthly for a maximum of nine examinations and were assigned inflammation scores for PLH, tracheal mucus, TW turbidity, and cell counts. Race place and failure to race were recorded for 327 horses in the study. Generalised estimating equation (GEE) models were used as repeated measure models for each risk factor controlling for other factors affecting race performance, such as the trainer, race value, and the horses’ age and gender. Increased PLH was associated with a decreased likelihood of racing, but not with race place. A moderate to severe increase in the tracheal mucus scores was associated with decreased likelihood of racing and also with increased race place. There was no significant association between TW cytology and racing or race place. In fact, the horses that raced had higher numbers of tracheal neutrophils than those that did not race, suggesting that this may be a normal adaptation to active training [39,40]. In South Africa, Saulez and Gummow [10] examined 1005 horses by endoscopic examination after racing and reported that tracheal mucous was detected in 99.5% of horses but they did not detect an association between mucous grades and race places or stake earnings. They also graded 372 horses for PLH and reported on a regression analysis, that a decreased number of wins and places in racehorses with PLH was probably due to fewer starts. The grade of PLH did not appear to affect performance.

Ivester et al. [24] investigated the association of BAL fluid cytology with the racing performance of 64 horses from eight stables. Performance data based upon speed and adjusted for race and track variants were extracted from an official database for Thoroughbred racing. Airway inflammation was classified as neutrophilic, mastocytic, or mixed, based on BAL fluid differential cytology counts. Mixed logistic regression models that controlled for age, trainer, and pulmonary haemorrhage, demonstrated a statistically significant negative impact of the mast cell and neutrophil proportions upon performance. This was also demonstrated for the increased absolute cell numbers. Mast cells appeared to have a stronger effect upon speed than neutrophils and were associated with a decreased likelihood of winning. Neutrophil cell proportions did not significantly affect the likelihood of winning.

### 3.4. Infectious and Non-Infectious Respiratory Agents

Of two studies focused on respiratory viruses, one had a low risk of bias [31] and one had a high risk of bias [32]. A study relating to atmospheric pollutants had a moderate risk of bias [33].

In a prospective observational study of 31 horses included in the larger study by Ivester et al. [24], performance as measured by speed figure and adjusted for age was not associated with the detection of viruses (equine herpesviruses 1, 2, 4 and 5, or equine rhinitis viruses A or B) or virus loads in nasopharyngeal and tracheal brushings or BAL samples [31]. In contrast, in a six month longitudinal serology study of 30 horses, seroconversion to equine rhinitis virus A was significantly associated with the subsequent failure to race [32]. Unfortunately, the possible role of a bacterial infection as a cofactor was not investigated in this study, and the authors noted that an association does not demonstrate causation.

One study presented evidence for an association between air quality and performance. Using a sample of 162 races and the principal component analysis strategy, Araneda and Cavada [33] demonstrated that atmospheric pollutants had a negative impact on the winning speed at a racetrack in Chile. In the study by Ivester et al. [24], mast cell inflammation was associated with respirable β-glucan exposures, probably from poor-quality hay, but not with respirable dust. BAL fluid neutrophilia was strongly associated with respirable dust.

### 3.5. Exercise-Induced Pulmonary Haemorrhage (EIPH)

Of the five studies that focused primarily on EIPH, three studies had a low risk of bias [26,29,30], one had a moderate risk of bias [28], and one had a high risk of bias [27].

In the clinical exercise study by McKane et al. [21], the percentage erythrocytes in BAL samples was positively correlated to the run time to fatigue and the arterial oxygen. However, only small amounts of haemorrhage were detected in the horses tested.

Two large cross-sectional studies carried out in Australia [29] and South Africa [30] concluded that EIPH impairs racing performance in Thoroughbred racehorses. The Australian study included 744 horses from the stables of 214 trainers competing in 202 races at four racecourses. The South African study included 886 racehorses competing at five racecourses. The studies were similarly designed to allow direct comparison of the results. The horses were enrolled prior to racing and examined endoscopically within two hours after the race. Three individuals blinded to the identity of the horse and their performance assessed the results of the examination on videotape and scored the severity of EIPH. Distance behind the winner, race earnings, and finishing position were used as the performance metrics. Multivariable regression modelling was employed to control potential confounding. Both studies concluded that adjusting for factors that could influence race performance, EIPH is associated with poorer performance. Horses with EIPH were less likely to win, finished farther behind the winner than did horses without EIPH and were less likely to be in the 90th percentile or higher for race earnings. In a more localised study the negative impact of EIPH was corroborated in racehorses from a single training yard where visible tracheal blood after strenuous exercise was associated with poor performance [25]. A further blinded study of 1567 horses at three Australian racecourses conducted under race day conditions and controlled for confounding variables supported the association of severe EIPH with impairment of racing performance [26]. However, in this population mild to moderate EIPH was not associated with inferior race day performance, in fact there was an association with superior performance over the final 400m compared with horses with no evidence of EIPH.

A study of 744 horses conducted over nine years reported that those with severe EIPH had fewer subsequent race starts, suggesting a negative impact on long-term performance [28]. However, the failure to race is not solely related to athletic performance, but may be related to management decisions and other factors. A longitudinal study of 822 racehorses at the Hong Kong Jockey Club indicated that the finding of EIPH on endoscopy did not affect the longevity of a horse’s racing career or its total number of race starts in comparison to horses that were not identified as having EIPH [27]. However, the finding that horses with severe EIPH were more likely to be retired was consistent with the study by Sullivan et al. [28].

### 3.6. Pleuropneumonia and Pulmonary Abscesses

Two studies related to lung infections had a moderate risk of bias [34,35].

Seltzer et al. [35] carried out a retrospective case series analysis to determine the prognosis for a return to racing after recovery from infectious pleuropneumonia. The majority of the 70 horses (61%) admitted to the hospital with pneumonia and pleural effusion raced after recovery, and more than 50% of these won at least one race. Ainsworth et al. [34] performed a similar retrospective study to determine the effect of pulmonary abscesses on racing performance. Seven out of twenty racehorses did not race after their discharge. The performance of the other 13 horses was not significantly different from their performance prior to illness if they had raced, or from the expected performance (starts percentile rank or SPR) of racehorses of similar age and sex.

### 3.7. Coughing

One study focused on coughing had a high risk of bias [36]. At a JRA facility, horses with no confounding upper respiratory tract abnormality were classified as coughing (n = 66) and non-coughing (n = 20), with nine healthy racehorses included as controls [36]. The existence of a racing career and the number of starts prior to examination were significantly higher in the non-coughing group than the coughing group. However, the authors suggested that this may have been confounded by age, as the mean age was significantly higher in the non-coughing group compared to the coughing group. The control group had no starts at the time of the study. Overall, it was not possible to conclude an association between coughing and impaired performance from the findings of this study.

## 4. Discussion

This scoping review identified 20 publications, which were analysed by the QUIPs tool. Overall studies relating to airway inflammation had a higher risk of bias than studies relating to EIPH and other disorders. Studies having a high risk of bias, as identified using the QUIPs tool, were included not just because they highlighted international interest in the investigation of poor performance in racehorses, but with a view to stimulating discussion about evidence gaps and the possibility of developing a more harmonised, strategic approach to such investigations globally. Many studies document the prevalence and causes of respiratory tract disorders in racing Thoroughbreds, but neglect to determine their relationship to performance. The evidence supporting the relationship between proposed diagnostic indicators and poor performance is variable. Three studies were performed over twenty years ago [21,22,35] and some of the techniques used for example microbiological evaluation of BAL are no longer accepted as standard practice [22]. Studies based on the examination of horses referred for respiratory disorders or poor performance suffer from a selection bias, whereas studies of horses after racing only generate data concerning the health status of horses that are fit to race and exclude those whose training programme has been interrupted as a result of poor performance. It has been suggested that as the finishing position within a race is usually determined by very small performance margins and the many factors affecting performance are complex, studies focused on the risk factors associated with poor performance should include large numbers of horses and employ sophisticated statistical tools [30]. However, this review attempted to include a range of studies including observational studies and those that lacked power due to small numbers of horses, lack of a control group, and failure to control for confounding factors. There is a need for experts in the field to develop high-level guidance for the design of controlled performance studies in Thoroughbred racehorses to collect comprehensive data that would enable an evidence-based approach for the interpretation of endoscopic and laboratory findings. Advanced planning to avoid confounding factors, a lack of appropriate controls, and an unclear epidemiological design would increase the benefits to other investigators.

The development of an objective and standardised measure of racehorse performance, which could be used to compare performances between horses of varying ages, abilities, disciplines, distances, and racing jurisdictions is undoubtedly challenging and has not been achieved to date. In the absence of such an ideal metric, a range of measures have been used in the studies of racehorse performance, and, in the studies reviewed here, categorical measures of starts/wins/places were most frequently used. The ability of a horse to return to racing post-illness or veterinary intervention is a fundamental measure of performance and is included in the former categorical measure. Indeed, it has been recommended that this measure be used in all clinical studies relating to performance [41]. Earnings-based measures and the finishing position were the second most frequently used performance metrics in the studies reviewed. While measures based on earnings are frequently used in performance-related studies, they are not without disadvantages, as there is no way to differentiate between the performance of horses that do not earn prizemoney. Earnings are also influenced by the quality of the race and the prizemoney available in each racing jurisdiction. While the use of several individual or categories of performance measure in a single study may give a more rounded view of the influence of various parameters on racehorse performance, it runs the risk of identifying significant differences in performance where none may truly exist.

For conditions such as mild to moderate asthma, formerly IAD, and EIPH, there appears to be a threshold below which they are unlikely to have a significant impact on performance. Indeed, the majority of racehorses appear to suffer repeated alveolar haemorrhage throughout their career due to the triggering of pulmonary vascular pressure by strenuous exercise [11,21]. In the clinical exercise study by Nolen-Watson et al. [18], most of the horses had evidence of a previous intrapulmonary haemorrhage, and 30% of the horses had erythrocytes present in BAL fluid after exercise. Mild to moderate asthma may affect up to 80–90% of racehorses [24,42], which could be indicative of a normal response to training or a reflection of the environment in which racehorses are maintained. The aetiology of mild to moderate asthma in horses is poorly defined and is considered to be multifactorial. Although infectious agents, particularly bacteria and to a lesser extent viruses, have been associated with asthma in Thoroughbred racehorses, the evidence is frequently controversial, particularly as several respiratory viruses are commonly detected in samples from healthy horses [31,43,44,45,46]. It may be that at least some infectious agents are not a cause of equine asthma but are opportunistic colonisers of already damaged airways [47]. However, recent infections with rhinovirus are implicated as the common causes of the induction and exacerbation of asthma in humans [48], and therefore, a connection of asthma in horses with equine rhinitis virus merits investigation [49]. Organic particulates are implicated as risk factors in the development of asthma; fungal exposure has been associated with increased mast cells in BAL fluid [24], respirable dust exposure has been associated with airway eosinophilia [8] and increased tracheal mucous [50], and exposure to pollen is associated with equine asthma exacerbation [51]. However, there are currently few studies that unequivocally support or refute a link between specific pathogens or environmental factors and poor performance in racehorses. Notwithstanding this knowledge gap, airborne contaminants in stables are similar to those associated with respiratory disease in humans, and the identification and correction of defects in ventilation, fodder, and bedding are universally accepted as promoting the optimum respiratory health in horses [5,8,52]. Published research findings substantiating a direct measurable performance improvement after such interventions would promote greater acceptance amongst trainers of the benefits of investing time and money in environmental management.

Given the increased trend for trainers to turn to veterinary interventions to enhance respiratory performance when racehorses present a poor performance, it is important that such interventions are evidence-based, with a strong focus on welfare. In particular, caution needs to be taken to safeguard welfare when considering that respiratory disease at certain thresholds may not impact performance. Normal ranges and thresholds for mild to moderate asthma and EIPH need to be determined by experienced racing clinicians from Australasia, Africa, the Americas, and Europe. In humans, asthma results in airway remodelling with an increase in airway smooth muscle [53]. Tissue remodelling of the bronchial lamina propria, epithelium, and smooth muscle was recently described in horses with mild and moderate asthma [54]. Future studies should address whether airway remodelling can be used to predict the progression of disease in the horse. Longitudinal studies of horses in training would facilitate the monitoring of asthma of varying immunological signatures and the outcome of interventions to prevent or reverse airway remodelling and inflammation. Similar longitudinal studies of EIPH are indicated to avoid long-term impacts of continuing to exercise, forced retirement, and the development of debilitating conditions such as pulmonary fibrosis, resulting from the repeated presence of blood in the lungs. It is essential that all potential confounders are measured in such longitudinal studies and that the statistical model selected is adequate to limit any possible bias.

The consequences of overdiagnosis have the potential to be as detrimental as those associated with underdiagnosis. “Lack of ability” has been described as the “worst problem of all” [55], and the unnecessary use of antibiotics, glucocorticoids, and other drugs in the face of owner disappointment, and the threat of financial loss to the trainer can jeopardise horse health. Furthermore, some problems are best solved by changes in management strategy rather than therapy. The recent outbreak of a highly contagious diarrhoea due to rotavirus B in Kentucky [56], has been largely controlled by foaling mares out of doors and minimising human contact. It may be that more innovative approaches to stabling and exposure of racehorses to respirable dust is required to minimise the risk of asthma and other respiratory conditions. Study design including appropriate controls needs to be considered prior to initiating environmental changes. This should ensure the delivery of high-quality evidence to inform recommendations that are readily accepted by all stakeholders.

As with all equestrian sport, there is an element of risk associated with racing. Defining safety risks to the horse and the jockey is an ongoing challenge for the industry, which has a responsibility to ensure that no injury or fatality occurs that could have reasonably been prevented. The indirect risk factors of impaired respiratory health in racehorses need to be identified, for example, the possibility of an association between respiratory stress and errors or falls in jump racing merits investigation. Standardising methodologies, improving investigation of poor performance, and stimulating discussion between stakeholders can support horse welfare in the future and contribute to the sector’s social license.

## 5. Study Limitations

The scoping review was conducted using three major electronic databases, Scopus, PubMed, and CAB direct. Additional relevant studies might have been identified had other databases been included. Broad search terms and inclusion criteria were used to capture as many of the appropriate studies as possible; however, this search strategy missed some studies later identified by citation searching. Furthermore, there was no date restriction used in the search and some of the older studies used out-dated terminology and methods that are no longer standard practice.

## 6. Conclusions

A scoping review of the available literature suggests that caution needs to be exercised when interpreting endoscopic and laboratory findings in the investigation of poor performance of Thoroughbred racehorses, as in many cases the functional relevance is difficult to establish. A high risk of bias was identified in several studies, predominantly relating to study confounding and statistical analysis and reporting. An improved study design and a harmonisation of approach to the assessment of poor performance would allow direct comparison between studies. A clearer understanding of aetiology would enable the implementation of evidence-based management strategies that would benefit the health, welfare, and performance of racehorses internationally.

## References

[1] Jeffcott L.B., Rossdale P.D., Freestone J., Frank C.J., Towers-Clark P.F. (1982). An assessment of wastage in thoroughbred racing from conception to 4 years of age. Equine Vet. J..

[2] Rossdale P.D., Hopes R., Digby N.J., Offord K. (1985). Epidemiological study of wastage among racehorses 1982 and 1983. Vet. Rec..

[3] Cardwell J.M., Smith K.C., Wood J.L., Newton J.R. (2014). Infectious risk factors and clinical indicators for tracheal mucus in British National Hunt racehorses. Equine Vet. J..

[4] Cardwell J.M., Smith K.C., Wood J.L., Newton J.R. (2013). A longitudinal study of respiratory infections in British National Hunt racehorses. Vet. Rec..

[5] Couëtil L.L., Cardwell J.M., Gerber V., Lavoie J.-P., Léguillette R., Richard E.A. (2016). Inflammatory Airway Disease of Horses—Revised Consensus Statement. J. Vet. Intern. Med..

[6] Kinnison T., Cardwell J.M. (2020). Conflict between Direct Experience and Research-Based Evidence Is a Key Challenge to Evidence-Based Respiratory Medicine on British Racing Yards. Front. Vet. Sci..

[7] Kinnison T., McGilvray T.A., Couëtil L.L., Smith K.C., Wylie C.E., Bacigalupo S.A., Gomez-Grau E., Cardwell J.M. (2022). Mild-moderate equine asthma: A scoping review of evidence supporting the consensus definition. Vet. J..

[8] Ivester K.M., Couëtil L.L., Moore G.E., Zimmerman N.J., Raskin R.E. (2014). Environmental exposures and airway inflammation in young thoroughbred horses. J. Vet. Intern. Med..

[9] Mazan M.R., Hoffman A.M. (2001). Effects of aerosolized albuterol on physiologic responses to exercise in standardbreds. Am. J. Vet. Res..

[10] Saulez M.N., Gummow B. (2009). Prevalence of pharyngeal, laryngeal and tracheal disorders in thoroughbred racehorses, and effect on performance. Vet. Rec..

[11] Hinchcliff K.W., Couetil L.L., Knight P.K., Morley P.S., Robinson N.E., Sweeney C.R., van Erck E. (2015). Exercise induced pulmonary hemorrhage in horses: American College of Veterinary Internal Medicine consensus statement. J. Vet. Intern. Med..

[12] Lyle C.H., Uzal F.A., McGorum B.C., Aida H., Blissitt K.J., Case J.T., Charles J.T., Gardner I., Horadagoda N., Kusano K. (2011). Sudden death in racing Thoroughbred horses: An international multicentre study of post mortem findings. Equine Vet. J..

[13] Tricco A.C., Lillie E., Zarin W., O’Brien K.K., Colquhoun H., Levac D., Moher D., Peters M.D.J., Horsley T., Weeks L. (2018). PRISMA Extension for Scoping Reviews (PRISMA-ScR): Checklist and Explanation. Ann. Intern. Med..

[14] Ouzzani M., Hammady H., Fedorowicz Z., Elmagarmid A. (2016). Rayyan-a web and mobile app for systematic reviews. Syst. Rev..

[15] Hayden J.A., van der Windt D.A., Cartwright J.L., Côté P., Bombardier C. (2013). Assessing bias in studies of prognostic factors. Ann. Intern. Med..

[16] Cochrane Quality in Prognosis Studies Tool. http://methods.cochrane.org/sites/methods.cochrane.org.prognosis/files/uploads/QUIPS%20tool.pdf.

[17] Page M.J., McKenzie J.E., Bossuyt P.M., Boutron I., Hoffmann T.C., Mulrow C.D., Shamseer L., Tetzlaff J.M., Akl E.A., Brennan S.E. (2021). The PRISMA 2020 statement: An updated guideline for reporting systematic reviews. BMJ.

[18] Nolen-Walston R.D., Harris M., Agnew M.E., Martin B.B., Reef V.B., Boston R.C., Davidson E.J. (2013). Clinical and diagnostic features of inflammatory airway disease subtypes in horses examined because of poor performance: 98 cases (2004-2010). J. Am. Vet. Med. Assoc..

[19] Kusano K., Ishikawa Y., Seki K., Kusunose R. (2008). Characteristic of inflammatory airway disease in Japanese thoroughbred racehorses. J. Equine Sci..

[20] Allen K.J., Tremaine W.H., Franklin S.H. (2006). Prevalence of inflammatory airway disease in National Hunt horses referred for investigation of poor athletic performance. Equine Vet. J..

[21] McKane S.A., Rose R.J., Evans D.L. (1995). Comparison of bronchoalveolar lavage findings and measurements of gas exchange during exercise in horses with poor racing performance. N. Z. Vet. J..

[22] Fogarty U., Buckley T. (1991). Bronchoalveolar lavage findings in horses with exercise intolerance. Equine Vet.-J.

[23] Holcombe S.J., Robinson N.E., Derksen F.J., Bertold B., Genovese R., Miller R., De Feiter Rupp H., Carr E.A., Eberhart S.W., Boruta D. (2010). Effect of tracheal mucus and tracheal cytology on racing performance in Thoroughbred racehorses. Equine Vet. J..

[24] Ivester K.M., Couëtil L.L., Moore G.E. (2018). An observational study of environmental exposures, airway cytology, and performance in racing thoroughbreds. J. Vet. Intern. Med..

[25] Salz R.O., Ahern B.J., Boston R., Begg L.M. (2016). Association of tracheal mucus or blood and airway neutrophilia with racing performance in Thoroughbred horses in an Australian racing yard. Aust. Vet. J..

[26] Crispe E.J., Lester G.D., Secombe C.J., Perera D.I. (2017). The association between exercise-induced pulmonary haemorrhage and race-day performance in Thoroughbred racehorses. Equine Vet. J..

[27] Preston S.A., Riggs C.M., Singleton M.D., Troedsson M.H. (2015). Descriptive analysis of longitudinal endoscopy for exercise-induced pulmonary haemorrhage in Thoroughbred racehorses training and racing at the Hong Kong Jockey Club. Equine Vet. J..

[28] Sullivan S.L., Anderson G.A., Morley P.S., Hinchcliff K.W. (2015). Prospective study of the association between exercise-induced pulmonary haemorrhage and long-term performance in Thoroughbred racehorses. Equine Vet. J..

[29] Hinchcliff K.W., Jackson M.A., Morley P.S., Brown J.A., Dredge A.E., O’Callaghan P.A., McCaffrey J.P., Slocombe R.E., Clarke A.E. (2005). Association between exercise-induced pulmonary hemorrhage and performance in Thoroughbred racehorses. J. Am. Vet. Med. Assoc..

[30] Morley P.S., Bromberek J.L., Saulez M.N., Hinchcliff K.W., Guthrie A.J. (2015). Exercise-induced pulmonary haemorrhage impairs racing performance in Thoroughbred racehorses. Equine Vet. J..

[31] Couetil L., Ivester K., Barnum S., Pusterla N. (2021). Equine respiratory viruses, airway inflammation and performance in thoroughbred racehorses. Vet. Microbiol..

[32] Back H., Weld J., Walsh C., Cullinane A. (2019). Equine rhinitis a virus infection in thoroughbred racehorses-a putative role in poor performance?. Viruses.

[33] Araneda O.F., Cavada G. (2022). Atmospheric Pollutants Affect Physical Performance: A Natural Experiment in Horse Racing Studied by Principal Component Analysis. Biology.

[34] Ainsworth D.M., Erb H.N., Eicker S.W., Yeagar A.E., Viel L., Sweeney C.R., Lavoie J.P. (2000). Effects of pulmonary abscesses on racing performance of horses treated at referral veterinary medical teaching hospitals: 45 cases (1985-1997). J. Am. Vet. Med. Assoc..

[35] Seltzer K.L., Byars T.D. (1996). Prognosis for return to racing after recovery from infectious pleuropneumonia in Thoroughbred racehorses: 70 cases (1984-1989). J. Am. Vet. Med. Assoc..

[36] Kusano K., Hobo S., Ode H., Ishikawa Y. (2008). Tracheal endoscopic and cytological findings and blood examination results in thoroughbred racehorses suspected to have lower respiratory tract disease. J. Equine Sci..

[37] Gerber V., Straub R., Marti E., Hauptman J., Herholz C., King M., Imhof A., Tahon L., Robinson N.E. (2004). Endoscopic scoring of mucus quantity and quality: Observer and horse variance and relationship to inflammation, mucus viscoelasticity and volume. Equine Vet. J..

[38] Raker C., Boles C.L. (1978). Pharyngeal lymphoid hyperplasia in the horse. J. Equine Med. Surg..

[39] McKane S.A., Canfield P.J., Rose R.J. (1993). Equine bronchoalveolar lavage cytology: Survey of thoroughbred racehorses in training. Aust. Vet. J..

[40] Bonsignore M.R., Morici G., Vignola A.M., Riccobono L., Bonanno A., Profita M., Abate P., Scichilone N., Amato G., Bellia V. (2003). Increased airway inflammatory cells in endurance athletes: What do they mean?. Clin. Exp. Allergy J. Br. Soc. Allergy Clin. Immunol..

[41] Wylie C.E., Newton J.R. (2018). A systematic literature search to identify performance measure outcomes used in clinical studies of racehorses. Equine Vet. J..

[42] Depecker M., Richard E.A., Pitel P.H., Fortier G., Leleu C., Couroucé-Malblanc A. (2014). Bronchoalveolar lavage fluid in Standardbred racehorses: Influence of unilateral/bilateral profiles and cut-off values on lower airway disease diagnosis. Vet. J..

[43] Christley R.M., Hodgson D.R., Rose R.J., Hodgson J.L., Wood J.L.N., Reid S.W.J. (2001). Coughing in thoroughbred racehorses: Risk factors and tracheal endoscopic and cytological findings. Vet. Rec..

[44] Burrell M.H., Wood J.L., Whitwell K.E., Chanter N., Mackintosh M.E., Mumford J.A. (1996). Respiratory disease in thoroughbred horses in training: The relationships between disease and viruses, bacteria and environment. Vet. Rec..

[45] Chapman P.S., Green C., Main J.P., Taylor P.M., Cunningham F.M., Cook A.J., Marr C.M. (2000). Retrospective study of the relationships between age, inflammation and the isolation of bacteria from the lower respiratory tract of thoroughbred horses. Vet. Rec..

[46] Newton J.R., Wood J.L.N., Chanter N. (2003). A case control study of factors and infections associated with clinically apparent respiratory disease in UK Thoroughbred racehorses. Prev. Vet. Med..

[47] Couetil L., Cardwell J.M., Leguillette R., Mazan M., Richard E., Bienzle D., Bullone M., Gerber V., Ivester K., Lavoie J.-P. (2020). Equine Asthma: Current Understanding and Future Directions. Front. Vet. Sci..

[48] Hansbro N.G., Horvat J.C., Wark P.A., Hansbro P.M. (2008). Understanding the mechanisms of viral induced asthma: New therapeutic directions. Pharmacol. Ther..

[49] Houtsma A., Bedenice D., Pusterla N., Pugliese B., Mapes S., Hoffman A.M., Paxson J., Rozanski E., Mukherjee J., Wigley M. (2015). Association between inflammatory airway disease of horses and exposure to respiratory viruses: A case control study. Multidiscip. Respir. Med..

[50] Millerick-May M.L., Karmaus W., Derksen F.J., Berthold B., Holcombe S.J., Robinson N.E. (2013). Local airborne particulate concentration is associated with visible tracheal mucus in Thoroughbred racehorses. Equine Vet. J..

[51] Costa M.F., Thomassian A. (2006). Evaluation of race distance, track surface and season of the year on exercise-induced pulmonary haemorrhage in flat racing thoroughbreds in Brazil. Equine Vet. J. Suppl.

[52] Dauvillier J., ter Woort F., van Erck-Westergren E. (2019). Fungi in respiratory samples of horses with inflammatory airway disease. J. Vet. Intern. Med..

[53] Hough K.P., Curtiss M.L., Blain T.J., Liu R.M., Trevor J., Deshane J.S., Thannickal V.J. (2020). Airway Remodeling in Asthma. Front. Med..

[54] Bessonnat A., Hélie P., Grimes C., Lavoie J.P. (2022). Airway remodeling in horses with mild and moderate asthma. J. Vet. Intern. Med..

[55] Wilsher S., Allen W.R., Wood J.L. (2006). Factors associated with failure of thoroughbred horses to train and race. Equine Vet. J..

[56] Uprety T., Sreenivasan C.C., Hause B.M., Li G., Odemuyiwa S.O., Locke S., Morgan J., Zeng L., Gilsenan W.F., Slovis N. (2021). Identification of a Ruminant Origin Group B Rotavirus Associated with Diarrhea Outbreaks in Foals. Viruses.

